# A Palmitoylethanolamide Producing *Lactobacillus paracasei* Improves *Clostridium difficile* Toxin A-Induced Colitis

**DOI:** 10.3389/fphar.2021.639728

**Published:** 2021-04-27

**Authors:** Giuseppe Esposito, Chiara Corpetti, Marcella Pesce, Luisa Seguella, Giuseppe Annunziata, Alessandro Del Re, Martina Vincenzi, Roberta Lattanzi, Jie Lu, Walter Sanseverino, Giovanni Sarnelli

**Affiliations:** ^1^ Department of Physiology and Pharmacology, “V. Erspamer”, Sapienza University of Rome, Rome, Italy; ^2^ Nextbiomics S.r.l., Naples, Italy; ^3^ Department of Clinical Medicine and Surgery, Section of Gastroenterology, University Federico II, Naples, Italy; ^4^ Department of Pharmacy, Faculty of Pharmacy, University Federico II, Naples, Italy; ^5^ Department of Human Anatomy, College of Basic Medical Sciences, China Medical University, Shenyang, China; ^6^ UNESCO Chair Staff Member, University of Naples “Federico II”, Naples, Italy

**Keywords:** palmitoylethanolamide, *Clostridium difficile* toxin A, colitis, *Lactobacillus paracasei*, next generation probiotics

## Abstract

Genetically engineered probiotics, able to *in situ* deliver therapeutically active compounds while restoring gut *eubiosis*, could represent an attractive therapeutic alternative in *Clostridium difficile* infection (CDI). Palmitoylethanolamide is an endogenous lipid able to exert immunomodulatory activities and restore epithelial barrier integrity in human models of colitis, by binding the peroxisome proliferator–activated receptor-α (PPARα). The aim of this study was to explore the efficacy of a newly designed PEA-producing probiotic (pNAPE-LP) in a mice model of *C. difficile* toxin A (TcdA)-induced colitis. The human N-acyl-phosphatidylethanolamine-specific phospholipase D (NAPE-PLD), a key enzyme involved in the synthesis of PEA, was cloned and expressed in a *Lactobacillus paracasei* that was intragastrically administered to mice 7 days prior the induction of the colitis. Bacteria carrying the empty vector served as negative controls (pLP).In the presence of palmitate, pNAPE-LP was able to significantly increase PEA production by 27,900%, in a time- and concentration-dependent fashion. Mice treated with pNAPE-LP showed a significant improvement of colitis in terms of histological damage score, macrophage count, and myeloperoxidase levels (−53, −82, and −70.4%, respectively). This was paralleled by a significant decrease both in the expression of toll-like receptor-4 (−71%), phospho-p38 mitogen-activated protein kinase (−72%), hypoxia-inducible factor-1-alpha (−53%), p50 (−74%), and p65 (−60%) and in the plasmatic levels of interleukin-6 (−86%), nitric oxide (−59%), and vascular endothelial growth factor (−71%). Finally, tight junction protein expression was significantly improved by pNAPE-LP treatment as witnessed by the rescue of zonula occludens-1 (+304%), Ras homolog family member A-GTP (+649%), and occludin expression (+160%). These protective effects were mediated by the specific release of PEA by the engineered probiotic as they were abolished in PPARα knockout mice and in wild-type mice treated with pLP. Herein, we demonstrated that pNAPE-LP has therapeutic potential in CDI by inhibiting colonic inflammation and restoring tight junction protein expression in mice, paving the way to next generation probiotics as a promising strategy in CDI prevention.

## Introduction

*Clostridium* (*Clostridioides*) *difficile* infection (CDI) represents the leading cause of nosocomial diarrhea in North America and Europe and has been labeled as an urgent public health threat by the US Center for Disease Control and Prevention (CDC) (Lessa et al., 2015). The disease almost invariably follows a disruption of gut resident flora, allowing *C. difficile* colonization and germination of the colon, commonly caused by broad-spectrum antibiotic use. Recent estimates indicate that *C. difficile* strains can be found in up to 50% of asymptomatic hospitalized patients; while in symptomatic individuals, the clinical spectrum may vary from uncomplicated diarrhea to even lethal pseudomembranous colitis, depending on strain virulence, on one hand and intestinal microecological conditions (competitive colonization resistance from host microflora), on the other (Napolitano and Edmiston, 2017).

*C. difficile* virulence depends on two bacterial exotoxins, *C. difficile* toxin A and B (TcdA and B, respectively) (Schäffler and Breitrück, 2018), that are internalized into the host cells via receptor-mediated endocytosis and inhibited by glycosylation Ras homolog family member A-GTPase (RhoA-GTPase) (Chen et al., 2015). RhoA-GTPase proteins are physiologically involved in actin cytoskeleton and tight junctions’ assembly (Terry et al., 2010), resulting in the disruption of the epithelial barrier, and consequently, a profound inflammatory response with release of pro-inflammatory cytokines and extensive neutrophil infiltration, through activation of the nuclear factor-kappa B (NF-κB) signaling pathway (Kim et al., 2006).

Palmitoylethanolamide (PEA) is an endogenous, on demand-released N-acylethanolamine belonging to the family of bioactive autacoid local injury antagonist amides (ALIAmides), a group of lipid molecules involved in the regulation of several physiological processes, ranging from analgesia, neuroprotection, and inflammation (Petrosino and Di Marzo, 2017). By selectively binding the peroxisome proliferator–activated receptor-α (PPARα) (Sarnelli et al., 2016b), PEA exerts a wide range of anti-inflammatory effects, downregulating inducible nitric oxide synthase (iNOS), cycloxigenase-2 (COX-2), tumor necrosis factor-α (TNF-α) expression, and the NF-κB signaling pathway, with downstream regulation of pro-inflammatory cytokines and immune cell infiltration in inflamed tissues. PEA anti-inflammatory effects have been tested in other animal models of colitis and in human-derived biopsies culture from ulcerative colitis patients. However, to date its potential in models of Clostridium difficile–induced colitis remains to be established (Esposito et al., 2014; Pesce et al., 2018).

PEA is produced by conjugation from palmitate and ethanolamine, through the N-acyl-phosphatidylethanolamine-specific phospholipase D (NAPE-PLD) (Pesce et al., 2018), a key enzyme in both ALIAmides and endocannabinoid synthesis and is rapidly degraded after binding its receptor targets. Owing to its on-demand activity, PEA is safe and virtually free from side effects but requires high doses to achieve significant pharmacological effects. From a translational standpoint, this unfavorable pharmacokinetic profile is a major setback in PEA translatability to clinical contexts. It is therefore pivotal to develop new formulations and/or delivery systems able to increase PEA tissue exposure, enhancing its contact surface in the attempt of achieving an efficient therapeutic response.

To overcome this limitation, we developed a probiotic-based delivery system, by genetically engineering *Lactobacillus paracasei subsp. paracasei* F19 with the human NAPE-PLD gene (p-NAPE-LP), in order to achieve an *in situ* delivery and release of PEA in the gastrointestinal tract, under the boost of ultralow doses of exogenous palmitate. *Lactobacilli* are able to survive the gastrointestinal tract and colonize the large intestine, where they constitute part of the endogenous microflora. They are recognized as safe (GRAS) for human consumption, making them suitable vehicles to deliver therapeutic molecules in the large intestine. Based on this background, we tested the efficacy of daily intragastric administration of p-NAPE-LP in preventing the severity of colitis induced by intrarectal instillation of TcdA, a well-validated murine model that resembles the most important features observed in CDI in humans (Markham et al., 2020).

## Materials and Methods

### Generation of Genetically Modified Strains of *Lactobacillus paracasei Subsp. Paracasei* F19 (pNAPE-LP)

The pTRKH3-slpGFP vector (Addgene, Watertown, Massachusetts, United States) was first modified to remove the GFP sequence at SalI/PstI restriction sites, insert the T7 transcriptional terminator at BamHI/EcoRV sites, and insert the linker sequence containing BsaI-BsaI at PstI/XmaI restriction sites. The cDNA of human NAPE-PLD was then inserted into the BsaI sites using In-Fusion method (Clontech, Mountain View, CA, United States). The resulting pTRKH3-slp-NAPE-PLD and parental plasmid (not expressing NAPE-PLD gene, used as negative control) constructs were transfected into the *L. paracasei subsp. paracasei* F19 strain (Arla Foods, Hoersholm, Denmark) by electroporation, and positive clones were obtained by erythromycin (5 μg/ml) selection. Both parental plasmid (pLP) and NAPE-PLD expressing bacteria (pNAPE-LP) were amplified anaerobically in Man, Rogosa, and Sharpe (MRS) broth (Conda, Torrejón de Ardoz Madrid, Spain) and isolated in MRS agar (Conda, Torrejón de Ardoz Madrid, Spain) both supplemented with erythromycin 5 μg/ml (Sigma-Aldrich, Milan, Italy) under anaerobic conditions for 72 h at 37°C. Bacteria viability was determined by manually counting colonies, and the colony forming units (CFU)/ml was obtained through a colony number correction for the dilution factor.

### Animals and Experimental Design

**SCHEME 1:**
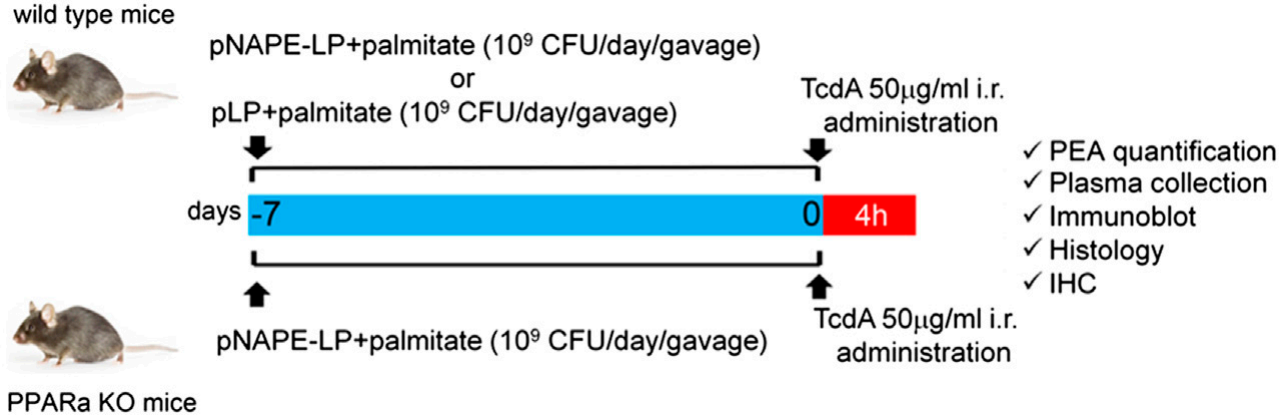
Experimental plan description: both wild type and PPARα KO mice received a daily prophylactic gavage administration of either pNAPE-LP (10^9^ CFU) or pLP (10^9^ CFU) 200 μl suspensions with sodium palmitate (0.0003 μg/ml). At day 7, animals received a single intrarectal instillation of TcdA (50 μg/ml). Animals were euthanized 4 h later and PEA quantification and other molecular/histological analyses were thus carried on postmortem isolated colonic tissue or related samples.

Six-week-old wild-type (WT) male C57BL/6J (Charles River, Laboratories, Italy) and PPARα knockout (KO) (Taconic, Germantown, New York, United States) mice were used for the experiments. All the procedures were approved by La Sapienza University's Ethics Committee in compliance with the IASP and European Community (EC L358/1 18/12/86) guidelines on the use and protection of animals in experimental research. As depicted in Scheme 1, C57BL/6J mice were randomly divided into four experimental groups (*n* = 10 per group): 1) vehicle (control group); 2) TcdA group; 3) TcdA + pLP group; and 4) TcdA + pNAPE-LP group, respectively. Both vehicle and TcdA groups received 200 μl MRS broth by intragastric gavage for one week, while the TcdA + pLP group was administered a daily volume of 200 μl of MRS broth suspension containing 10^9^ CFU of pLP strain with 0.0003 μg/ml of sodium palmitate (Sigma-Aldrich, Milan, Italy). In the same conditions, the TcdA + pNAPE-LP group was administered by intragastric gavage with a daily volume of 200 μl of MRS broth suspension containing 10^9^ CFU of pNAPE-LP and 0.0003 μg/ml of sodium palmitate. PPARα KO mice were randomly divided into three experimental groups (*n* = 6 per group): 1) vehicle (control group); 2) TcdA group; and 3) TcdA + pNAPE-LP group, respectively, and treated as above. One week following probiotic administration, mice received by intrarectal route, a single administration of phosphate buffered saline 1× (PBS 1×) or TcdA (50 μg/ml dissolved in PBS 1×), according to the method described by Hirota et al. (2012) to induce acute pseudomembranous colitis. Since animals’ death was not an acceptable experimental end point, based on the Institutional Animal Care and Use Committee (which ethically prevents the death of the animal as a terminal event of the procedure), we used a nonlethal dose of TcdA, and after 4 h from the intrarectal administration, all mice used for the experimental plan were deeply anesthetized before being euthanatized, as described by Hirota et al. (2012). Blood samples were collected by intracardiac puncture, and tissues were isolated and processed to further analyses (see below).

### Extraction and Quantification of *In Vitro* and *In Vivo* Produced PEA by HPLC–MS Method

Extraction and analysis of PEA released *in vitro* and *in vivo* were performed according to Gachet et al. (Gachet et al., 2015), with slight modifications. Bacterial cultures were ultracentrifuged at 14,000 rpm for 10 min to separate the culture medium and bacterial pellet. Culture media were freeze-dried and then resuspended in a solution containing acetonitrile with 0.1% formic acid (extraction solution). Then, the samples were ultracentrifuged (14,000 rpm, 4°C, 5 min) and the supernatant injected for the mass spectrometry analysis. Both bacterial pellet and mice colonic tissues were firstly lysed using a lysis buffer and then evaporated under a nitrogen stream. Residues were suspended in extraction solution, ultra-centrifuged (14,000 rpm, 4°C, 5 min), and the supernatant injected for the mass spectrometry analysis. Analyses were run on a Jasco Extrema LC-4000 system (Jasco Inc., Easton, MD) coupled to an Advion Expression mass spectrometer (Advion Inc., Ithaca, NY) equipped with an electrospray (ESI) source. Mass spectra were recorded in the positive SIM mode. The capillary voltage was set at +180V, the spray voltage was at 3 kV, the source voltage offset was at +20 V, and the capillary temperature was set at 250°C. The chromatographic separation was performed on analytical column Kinetex C18 (150 × 4.6 mm, id.3 µm, 100 Å) and security guard column both supplied by Phenomenex (Torrance, CA, United States). The analyses were performed at a flow rate of 0.3 ml/min, with solvent A (water containing 2 mM ammonium acetate) and solvent B (methanol containing 2 mM ammonium acetate and 0.1% formic acid). Elution was performed according to the following linear gradient: 15% B for 0.5 min, 15–70% B from 0.5 to 2.5 min, 7–99% B from 2.5 to 4.0 min, and held at 99% B from 4.0 to 8.0 min. From 8 to 11.50 min , the column was equilibrated to 15% B and conditioned from 11.5 to 15.0 at 15% B. The injection volume was 10 µl, and the column temperature was fixed at 40°C. For quantitative analysis, standard curves of PEA (Sigma-Aldrich, Milan, Italy) were prepared over a concentration range of 0.0001–10 ppm with six different concentration levels and duplicate injections at each level. All data were collected and processed using JASCO ChromNAV (version 2.02.04) and Advion Data Express (4.0.13.8).

### Histopathological Analysis

After sacrifice, mice distal colon specimens were fixed in 4% paraformaldehyde (PFA), sectioned into 15 μm slices, and stained with hematoxylin and eosin (H and E) (Sigma-Aldrich, Milan, Italy) for macroscopic and histopathological assessment. Colonic histological damage was evaluated through a complex score, according to the criteria proposed (Li et al., 2017), considering the following parameters: 1) distortion and loss of crypt architecture (0 = none; 1 = mild; 2 = moderate; 3 = severe); 2) infiltration of inflammatory cells (0 = normal; 1 = mild infiltration; 2 = moderate infiltration; 3 = dense infiltration); 3) muscle thickening (0 = normal; 1 = mild muscle thickening; 2 = moderate muscle thickening; 3 = marked muscle thickening); 4) goblet cells depletion (0 = absence; 1 = presence); and 5) crypt absence (0 = absence; 1 = presence). Slices were analyzed with a microscope Optika XDS-3L4 Ponteranica, BG, Italy), and images were captured at 4× magnification by a high-resolution digital camera (Nikon Digital Sight DS-U1). The cumulative histological damage score was expressed as average scores in each experimental group derived by the observations of two independent qualified observers (CC and GE).

### Determination of Macrophages Mucosal Infiltration

Samples for immunohistochemical assessment of macrophages were isolated from both WT and PPARα KO mouse distal colon. Tissues were fixed in 4% PFA, embedded in paraffin, sectioned in 15 μm slices, and processed for immunohistochemistry. Slices were pretreated using heat-mediated antigen retrieval with a sodium citrate buffer, incubated with MAC387 (Abcam, Cambridge, United Kingdom) at room temperature (RT) (Thoree et al., 2008), and detected using the horseradish peroxidase (HRP)–conjugated compact polymer system. 3,30-diaminobenzidine (DAB) was used as the chromogen. Slices were then counterstained with hematoxylin, mounted with Eukitt (Sigma-Aldrich, Milan, Italy), and analyzed with a microscope (Optika XDS-3L4 Ponteranica, BG, Italy). Images were captured at 10× with a high-resolution digital camera and the data represent the median results of the two blinded assessors (CC and GE); in all cases, results of the assessments differed by no more than 5%. Results were quantified by ImageJ software (National Institutes of Health) and expressed as the number of macrophage marker antibody (MAC387) positive cells per area.

### Myeloperoxidase Assay

Myeloperoxidase (MPO), a marker of polymorphonuclear leukocyte accumulation, was determined as previously described (Mullane et al., 1985). After removal, colonic tissues from both WT and PPARα KO mice were rinsed with a cold saline solution, opened, and deprived of the mucosa using a glass slide. The resulting layer was then homogenized in a solution containing 0.5% hexadecyltrimethylammonium bromide (Sigma-Aldrich, Milan, Italy), dissolved in 10 mM potassium phosphate buffer, and centrifuged for 30 min at 20,000 × g at 37°C. An aliquot of the supernatant was mixed with a solution of tetramethylbenzidine (1.6 mM; Sigma-Aldrich, Milan, Italy) and 0.1 mM hydrogen peroxide (Sigma-Aldrich, Milan, Italy). The solution was then spectrophotometrically measured at 650 nm. MPO activity was determined as the amount of enzyme degrading 1 mmol/min of peroxide at 37°C and was expressed in milliunits (mu) per 100 mg of wet tissue weight.

### Protein Extraction and Western Blot Analysis

Proteins were extracted from colonic tissue or bacteria pellets and processed by Western blot analysis. For protein extraction by the bacterial pellet, a specific CelLytic™ lysis buffer (Sigma-Aldrich, Milan, Italy) was used according to manufacturer’s instructions. Tissue samples were homogenized in ice-cold hypotonic lysis buffer [10 mM 4-(2-hydroxyethyl)-1-piperazineethanesulfonic acid (HEPES), 1.5 mM MgCl_2_, 10 mM KCl, 0.5 mM phenylmethylsulphonylfluoride, 1.5 μg/ml soybean trypsin inhibitor, 7 mg/ml pepstatin A, 5 mg/ml leupeptin, 0.1 mM benzamidine, and 0.5 mM dithiothreitol (DTT)]. Both bacterial- and tissue-deriving protein extracts were mixed with a nonreducing gel loading buffer [50 mM Tris (hydroxymethyl)aminomethane (Tris), 10% sodium dodecyl sulfate (SDS), 10% glycerol, 2 mg/ml bromophenol] at a 1:1 ratio, and then boiled for 3 min followed by centrifugation at 10,000×*g* for 10 min. The protein concentration was determined using Bradford assay and equivalent amounts (50 μg) of each homogenate underwent electrophoresis through a polyacrilamide minigel. After the transfer the membranes were incubated with 10% nonfat dry milk in PBS overnight at 4°C and then exposed, depending on the experiments, with rabbit polyclonal anti-NAPE-PLD (Abcam, Cambridge, United Kingdom) (1:200 v/v), rabbit polyclonal anti–toll-like receptor-4 (TLR4) (Bioss Antibodies, Boston, United States) (1:1,000 v/v), mouse monoclonal anti-RhoA-GTPase (Santa Cruz Biotechnology, Santa Cruz, CA, United States) (1:100 v/v), rabbit polyclonal anti-p38 mitogen-activated protein kinase (p38 MAPK) (Bioss Antibodies, Boston, United States (1:1,000 v/v), rabbit monoclonal anti-phospho-p-38 (p-p38) MAPK (Santa Cruz Biotechnology, Santa Cruz, CA, United States) (1:1,000 v/v), rabbit polyclonal anti-NF-κB p65 (Sigma-Aldrich, Milan, Italy) (1:1,000 v/v), mouse monoclonal anti-NF-κB p50 (Santa Cruz Biotechnology, Santa Cruz, CA, United States) (1:1,000 v/v), mouse monoclonal anti–hypoxia-inducible factor-1-alpha (HIF-1α) (Novus biological, Abingdon, United Kingdom) (1:500 v/v), and rabbit polyclonal anti-glyceraldehyde 3-phosphate dehydrogenase (GAPDH) (Cell Signaling Technology, Danvers, MA, United States) (1:1,000 v/v) according to standard experimental protocols. Membranes were then incubated with the specific secondary antibodies conjugated to HRP (Dako, Milan, Italy). Immune complexes were exposed to enhanced chemiluminescence detection reagents, and the blots were analyzed by scanning densitometry (Versadoc MP4000; Bio-Rad, Segrate, Italy). Results were expressed as optical density (OD; arbitrary units = mm^2^) and normalized against the expression of the housekeeping protein GAPDH. Immune complexes were revealed by enhanced chemiluminescence detection reagents (Amersham Biosciences, Milan, Italy) and exposed to the Kodak X-Omat film (Eastman Kodak Co., Rochester, NY, United States OK). Protein bands were then scanned and densitometrically analyzed with a GS-700 imaging densitometer. Results were expressed as OD (arbitrary units; mm^2^) and normalized on the expression of the housekeeping protein GAPDH for mice and proteins.

### Blood Samples Preparation

Before being sacrificed, mice were deeply anesthetized. Blood samples were taken by cardiac puncture and collected in 5% ethylenediaminetetraacetic acid (EDTA) vials, immediately prior to sacrifice. To determine nitric oxide (NO), interleukin-6 (IL-6), and vascular endothelial growth factor (VEGF) levels, plasma was then isolated from the blood, immediately frozen, and stored at −80°C until the assays.

### Enzyme-Linked Immunosorbent Assay for IL-6 and VEGF

Enzyme-linked immunosorbent assay (ELISA) for IL-6 and VEGF (all from Thermo Fisher Scientific Inc., Monza, Italy) was carried out on mice plasma according to the manufacturer’s protocol. Absorbance was measured on a microtiter plate reader. IL-6 and VEGF levels were determined using the standard curves method.

### NO Quantification

NO production was measured as nitrite (NO_2_^−^) accumulation in murine plasma by a spectrophotometer assay based on the Griess reaction (Di Rosa et al., 1990). Briefly, Griess reagent (1% sulfanilamide and 0.1% naphthylethylenediamine in H_3_PO_4_) was added to an equal volume of plasma, and the absorbance was measured at 550 nm. NO_2_^−^ concentration (nm) was thus determined using a standard curve of NaNO_2_.

### Immunofluorescence Analysis for Mucosal ZO-1 and Occludin

Segments of distal mouse colon were isolated and fixed in ice-cold 4% PFA and sectioned into 20 μm slices. Sections were thus blocked with bovine serum albumin and subsequently stained with mouse anti–zonula occludens-1 (ZO-1) antibody (Bioss Antibodies, Boston, United States) (1:100 v/v) or rabbit anti-occludin antibody (Novus biological, Abingdon, United Kingdom) (1:100 v/v). Slices were then washed with PBS 1× and incubated in the dark with fluorescein isothiocyanate–conjugated anti-rabbit (Abcam, Cambridge, United Kingdom). Nuclei were stained with 2-(4-amidinophenyl)-1H -indole-6-carboxamidine (DAPI) (Thermo Fisher Scientific, Massachusetts, United States). Sections were analyzed with a microscope (Optika XDS-3FL4 Ponteranica, BG, Italy), and images were captured by a high-resolution digital camera (Nikon Digital Sight DS-U1). The expression of zonula occludens (ZO-1) and occludin was measured as relative fluorescence units (RFU) fold change vs. vehicle groups.

### Statistical Analysis

Results are expressed as the mean ± standard error (SEM) of n sets of experiments in triplicate (see figure legends). Statistical analyses were performed using one-way analysis of variance, and multiple comparisons were performed using a Bonferroni post hoc test. *p < 0.05, **p < 0.01, and ***p < 0.001 were considered to indicate a statistically significant difference vs. control group, and °*p* < 0.05, ^oo^ *p* < 0.01, and ^ooo^ *p* < 0.001 were considered to indicate a statistically significant difference vs. TcdA group.

## Results

### Time- and palmitate concentration-dependent NAPE-PLD expression and PEA release by pNAPE-LP engineered bacteria *in vitro*

**FIGURE 1:**
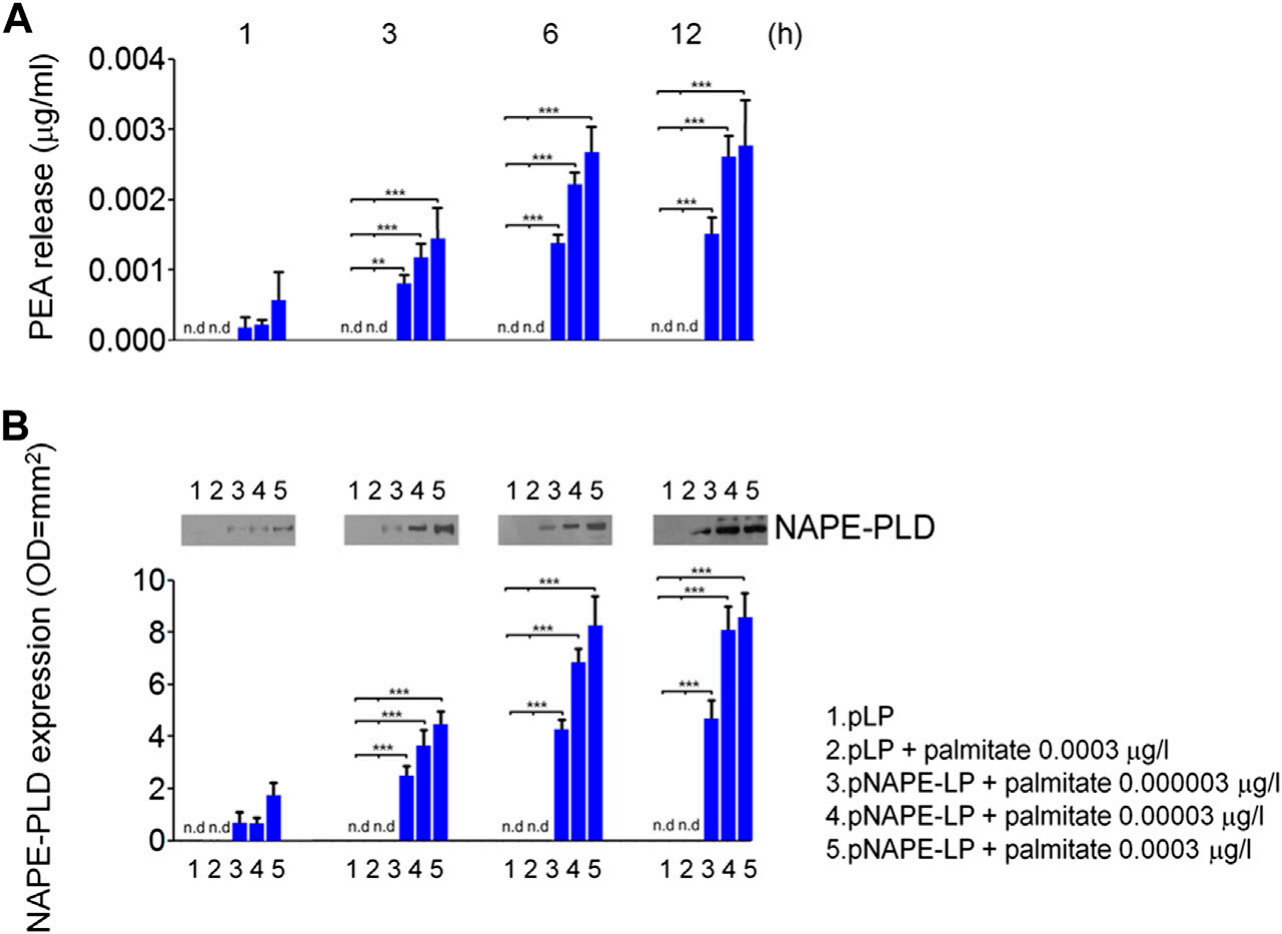
PEA is released *in vitro* by engineered NAPE-PLD *Lactobacillus paracasei* under palmitate dose and time dependent boost. **(A)** PEA release was evaluated in bacterial supernatants at 1, 3, 6, and 12 h, respectively, by HPLC–MS, and the results are expressed as mean ± SEM of *n* = 4 experiments performed in triplicate. Compared with pLP in absence of palmitate supply, exogenous palmitate (0.000003–0.0003 μg/l) dose- and time-dependently increased PEA release from pNAPE-LP probiotics, ***p < 0.001, **p < 0.01 vs. both pLP and pLP in presence of palmitate 0.0003 μg/l. PEA levels were undetectable in pLP supernatants, even in the presence of the highest tested doses of exogenous palmitate (0.0003 μg/l). In the same conditions, **(B)** Western blot analysis of NAPE-PLD expression and relative densitometric analysis of immunoreactive bands show that NAPE-PLD protein expression is time (1, 3, 6, and 12 h) and palmitate concentration (0.000003–0.0003 μg/l) dependent in pNAPE-LP engineered bacteria, whereas no expression was noticeable in pLP alone at the different time points, even in the presence of the highest palmitate doses (0.0003 μg/l). ***p < 0.001 vs. both pLP and pLP + palmitate 0.0003 μg/l n. d., non-detectable.

We first evaluated *in vitro* the ability of pNAPE-LP engineered strains to release PEA in the bacterial supernatant under the boost of the ultralow dose of exogenous palmitate and tested the optimal palmitate concentrations to use in our *in vivo* experiments. Our results demonstrated that NAPE-PLD protein expression increased in a time-dependent manner in pNAPE-LP bacteria, following culture medium supplementation with 0.000003–0.0003 μg/ml of palmitate, reaching an expression peak between 6 and 12 h and a plateau at 12 h (+89,000% vs. pLP) (Figures 1A,B), while NAPE-PLD was not detected in native pLP at the same time intervals. PEA concentrations significantly increased in the supernatant of pNAPE-LP, mirroring NAPE-PLD expression in a time and palmitate-dependent manner. The PEA level peaked at 12 h (+27900% vs. pLP) and reached a plateau concentration at the same time interval (Figures 1A,B). As anticipated, PEA levels were undetectable in pLP, even in the presence of the highest palmitate concentrations (0.0003 μg/l) (Figures 1A, B).

### *In vivo* NAPE-PLD Expression and PEA Release by pNAPE-LP Engineered Bacteria

At sacrifice, NAPE-PLD protein expression and PEA release were also evaluated in mice colonic tissues from the different experimental groups. Our data show that TcdA challenge, per se, increased NAPE-PLD expression and PEA release (+336% and +400%, respectively, vs. vehicle), while the treatment with native pLP + palmitate 0.0003 μg/ml led to a further but not significant increase in NAPE-PLD expression and PEA release (+21 and +8.95%, respectively, vs. TcdA group).

**FIGURE 2:**
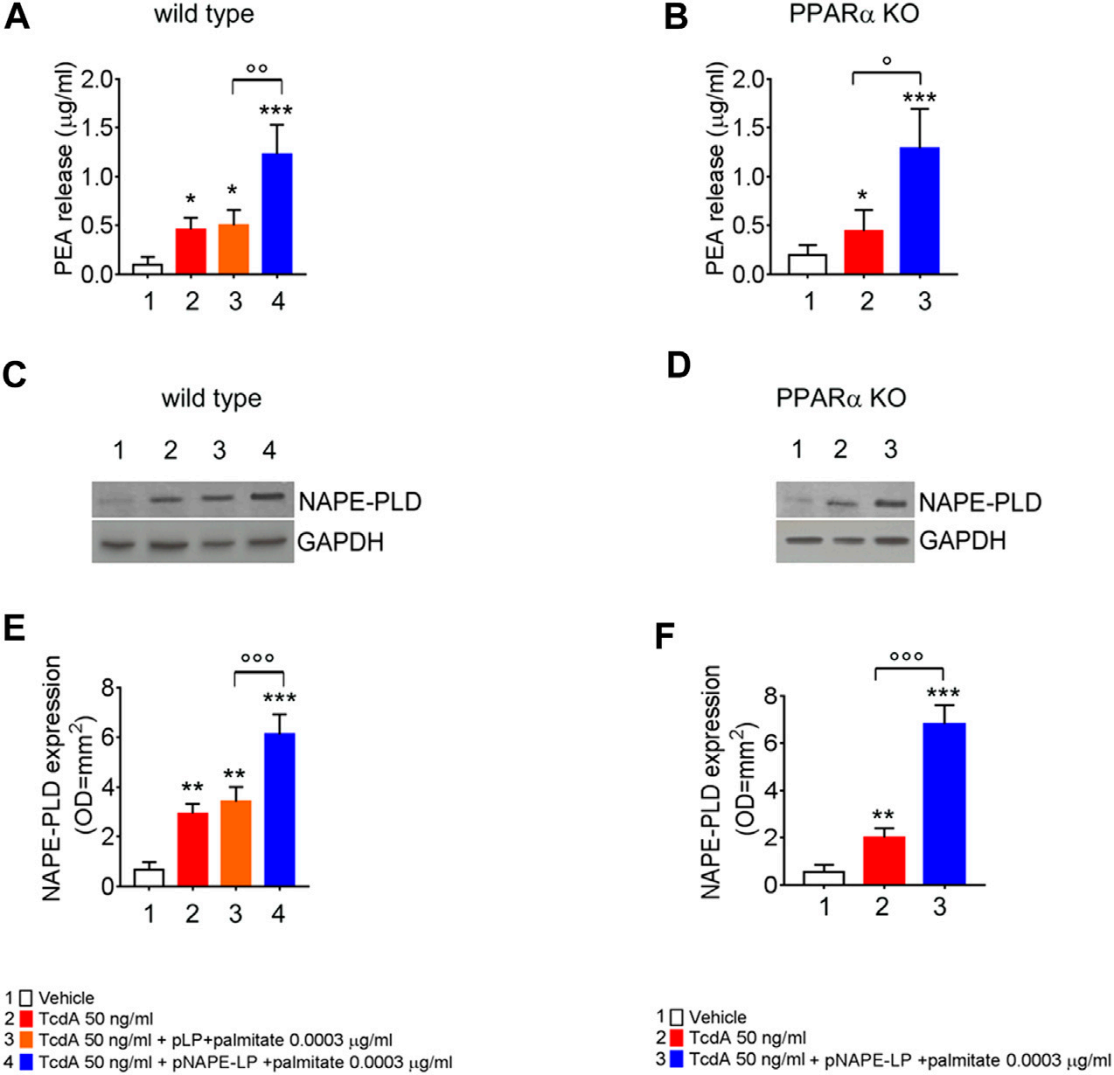
PEA is released *in vivo* by engineered NAPE-PLD probiotic under palmitate dose- and time-dependent boost. PEA levels were measured in both wild type **(A)** and PPARα KO mice **(B)** colon by HPLC–MS, and results are expressed as mean ± SEM of *n* = 6 experiments performed in triplicate. TcdA challenge caused PEA increase in both mice types (*p < 0.05 vs. respective controls). Figures **(A)** and **(B)** show that pNAPE-LP + palmitate (0.0003 μg/kg), resulted in a significantly increased PEA release as compared to vehicle (both ***p < 0.001 vs. vehicle) and TcdA-treated groups in both wild type and PPARα KO mice (^ooo^ *p* < 0.001 and ^oo^ *p* < 0.01 and °*p* < 0.05 vs. respective TcdA groups). Figure also shows Western blot analysis of NAPE-PLD expression and relative densitometric analysis of immunoreactive bands in both wild type **(C)** and PPARα KO mice **(D)** colon and their relative densitometric quantification **(E, F)**. Results are expressed as mean ± SEM of *n* = 6 experiments performed in triplicate. The TcdA challenge caused an increased expression of NAPE-PLD in both mice types (**p < 0.01 vs. respective controls). pNAPE-LP and palmitate (0.0003 μg/kg) supply resulted in a significantly higher NAPE-PLD protein expression in the colon of both untreated mice types (both ***p < 0.001 vs. vehicle) **(C–F)**, and in both wild type and PPARα KO mice treated with TcdA (both ^ooo^ *p* < 0.001 vs. respective TcdA groups).

Conversely, the treatment with pNAPE-LP + palmitate 0.0003 μg/ml resulted in a significant +85 and +72% relative increase of NAPE-PLD expression vs. TcdA and pLP + palmitate 0.0003 μg/ml treated mice, respectively. In line with NAPE-PLD expression, PEA concentrations in colonic specimens from pNAPE-LP + palmitate 0.0003 μg/ml treated mice were increased up to +1233% vs. vehicle and +150% vs. pLP + palmitate 0.0003 μg/ml, respectively, treated groups (Figures 2A,C,E).

### Treatment with pNAPE-LP and Palmitate Improves Colonic Histopathological Damage, Macrophage Density, and Neutrophil Infiltration in WT Mice

**FIGURE 3:**
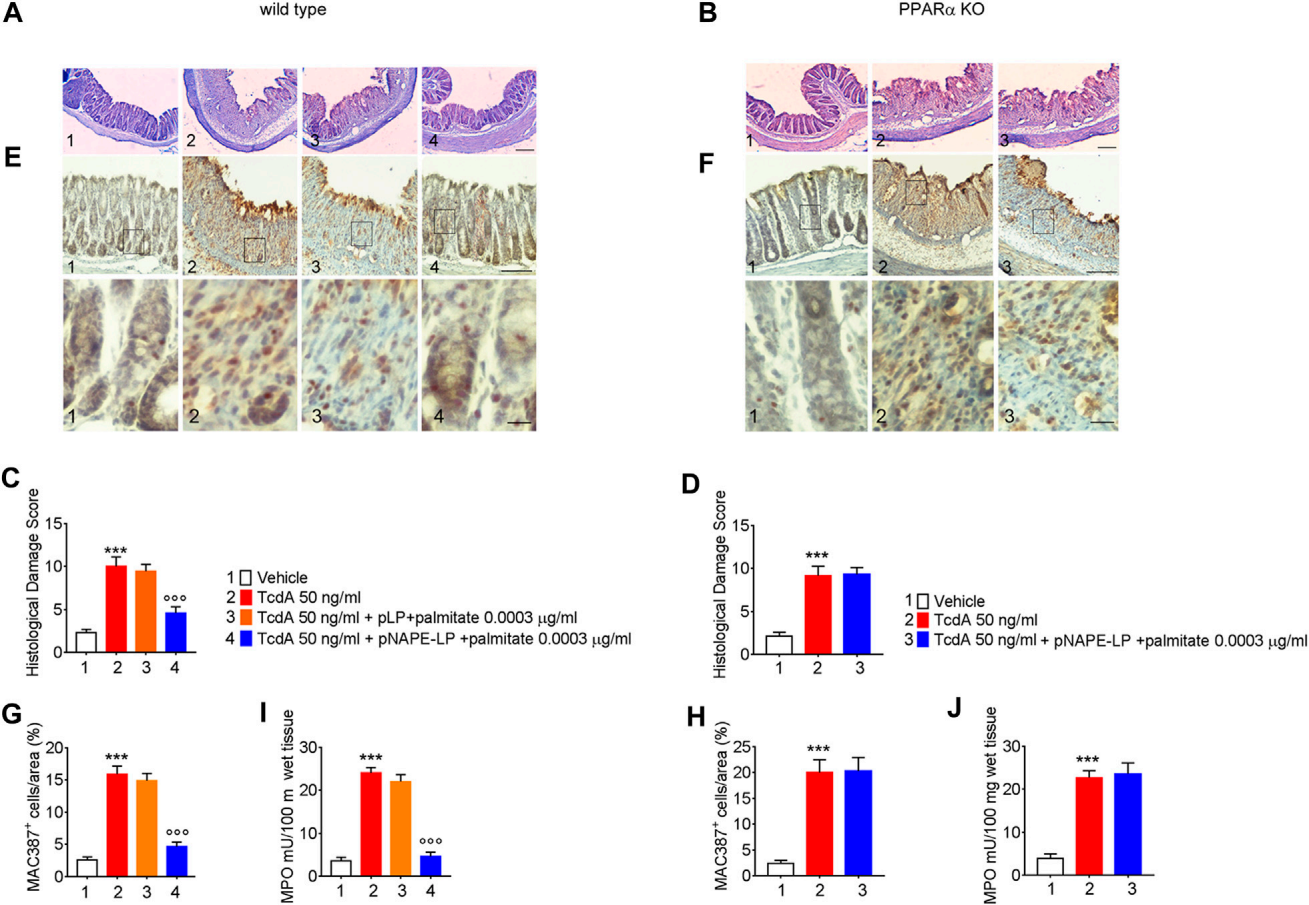
Prophylactic administration of pNAPE-LP and ultralow palmitate dose accounts for histological damage attenuation with macrophage and neutrophils infiltration reduction in TcdA challenged mice. Hematoxylin and eosin (H and E) stained distal colonic specimens and **(A, B)** relative histological damage score showing the protective and PPARα-dependent effect of pNAPE-LP/palmitate treatment on TcdA-induced colonic injury **(C, D)** in both wild type and PPARα mice (magnification 4×, scale bar: 100 μm). Figure also shows the effect of pNAPE-LP/palmitate association on the immunohistochemical expression of MAC387 positive cell (marker of macrophage density) in distal colonic sections deriving from wild type and PPARα KO mice **(E, F)** (magnification 10× and 40×, scale bar: 100 and 50 μm, respectively) and its relative quantification **(G, H)**, and the myeloperoxidase (MPO) activity quantification (indirect evidence of neutrophils infiltration) in both mice types **(J, K)**. Results are expressed as mean ± SEM of *n* = 5 experiments. ***p < 0.001 vs. vehicle; ^ooo^ *p* < 0.001 vs. TcdA-treated mice.

The TcdA challenge induced a severe mucosal damage evaluated at histopathological analysis performed by H and E (Figures 3A,C) in WT mice (+350% vs. vehicle). Mucosal inflammation was featured by a markedly increased macrophage density in the colonic mucosa, as per immunohistochemical quantification of MAC387 positive cells (+450% vs. vehicle) and by increased neutrophils infiltration, indirectly confirmed by the increased MPO activity (+633% vs. vehicle) (Figures 3E,G,H). A negligible and not significant improvement in terms of histological score (−6%), relative MAC387 density (−5%), and MPO activity (−9%) was observed in mice treated with native pLP + palmitate 0.0003 μg/ml. On the contrary, pNAPE-LP + palmitate 0.0003 μg/ml administration significantly improved the histological damage score (−53%), with a consequent reduction of MAC387 + cell count (−70.4%) and a significant reduction in MPO levels (−82%) compared to the TcdA group in WT mice.

### Treatment with pNAPE-LP and Palmitate Decreases Pro-Inflammatory Markers Expression and Cytokine Release in TcdA-Treated WT Mice

**FIGURE 4:**
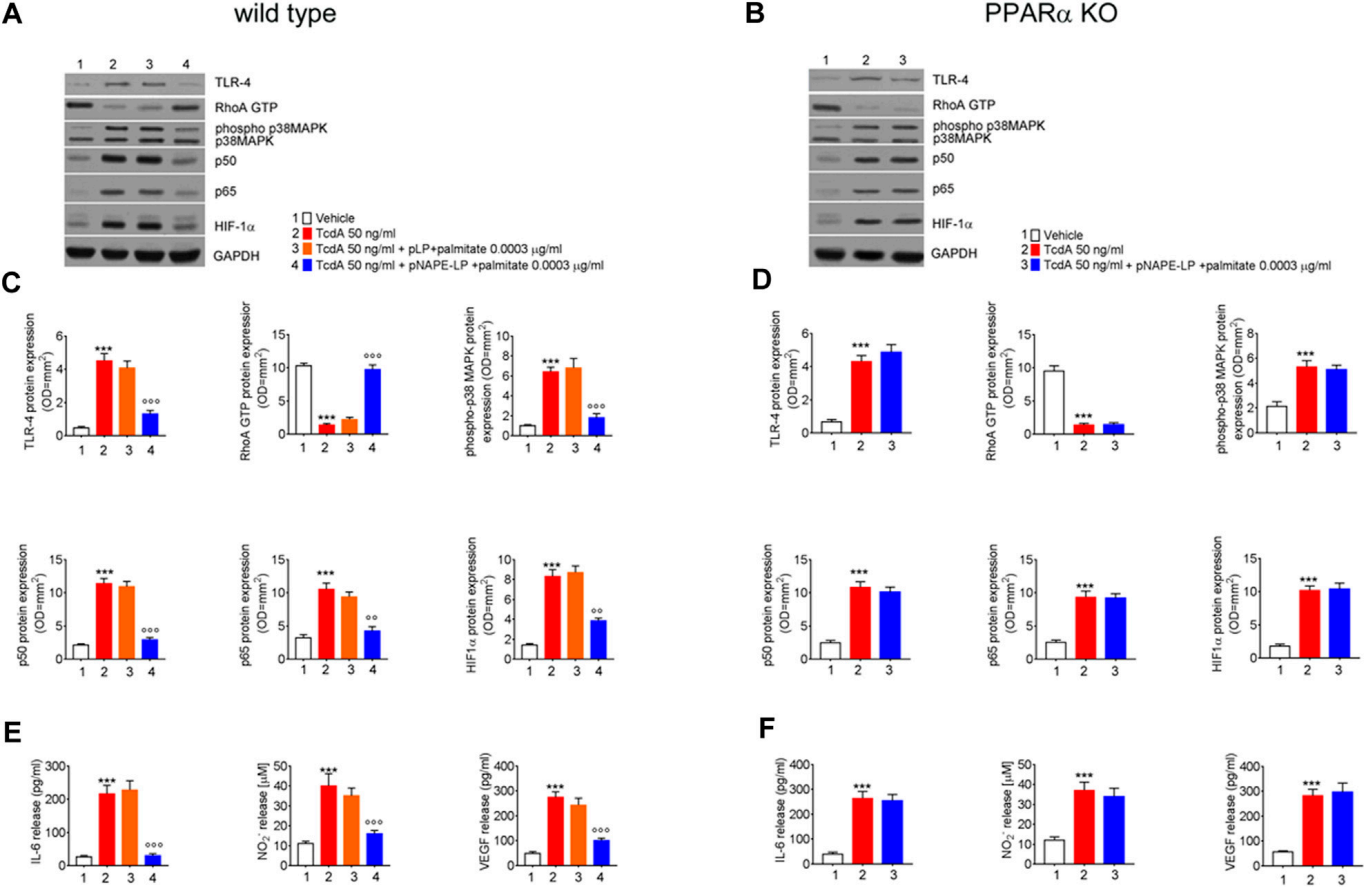
Prophylactic administration of pNAPE-LP and ultralow palmitate administration on pro-inflammatory signaling molecules expression and release in mice colon. Immunoreactive bands showing TLR-4, RhoA GTP, phosphorylated/unphosphorylated - p38 MAPK, NF-κB-related p50 and p65, and HIF-1α protein expression in both wild type **(A)** and PPARα KO **(B)** mice following TcdA challenge and their relative PPARα-dependent decrease following pNAPE-LP/palmitate co-administration. Relative densitometric analysis **(C**, **D)** of each protein (arbitrary units normalized on the expression of the housekeeping protein GAPDH). Results were expressed as mean ± SEM of *n* = 6 experiments performed in triplicate. ***p < 0.001 vs. vehicle; and ^ooo^ *p* < 0.001 and ^oo^ *p* < 0.01 vs. TcdA. The figure also shows the effect of pNAPE-LP on TcdA challenged wild type **(E)** and PPARα KO mice **(F)** in terms of release of IL-6, nitric oxide (NO), and VEGF in the plasma. Results were expressed as mean ± SEM of *n* = 6 experiments performed in triplicate. ***p < 0.001 vs. vehicle; and ^ooo^ *p* < 0.001 vs. TcdA.

The expression of pro-inflammatory signaling molecules and their release were evaluated in colonic tissue homogenates and plasma samples, respectively. Our results demonstrated that the intrarectal TcdA challenge caused a marked increase in the protein levels of TLR-4 (881%), phospho-p38 MAPK (550%), HIF1α (+489%), and of the markers of NF-κB activation p50 (433%) and p65 (230%), vs. vehicle in C57BL/6 J mice. Immunoblot analysis also revealed a massive decrease of RhoA-GTPase protein expression in TcdA vs. vehicle group (−87%) (Figures 4A,C). In parallel, plasmatic levels of IL-6 (+740%), NO (+245%), and VEGF (458%) were significantly increased in TcdA as compared to vehicle (Figure 4E).

pLP + palmitate 0.0003 μg/ml coadministration failed to improve the above-described parameters, as we observed a not significant variation in TLR-4 (−10%), phospho-p38 MAPK (+6%), HIF-1α (+5%) p50 (−4.0%), p65 (-10%), and RhoA-GTPase (+9.5%) protein expression. Similarly, plasmatic pro-inflammatory mediators such as IL-6 (+5.6%), NO (−10%), and VEGF (−11%) were not significantly improved by native pLP + palmitate 0.0003 μg/ml vs. TcdA group.

Conversely, in the group of WT mice treated with pNAPE-LP + palmitate 0.0003 μg/ml, we observed a reduced expression of pro-inflammatory signaling molecules with a significant decrease of TLR-4 (−71%), phospho-p38 MAPK (−72%), HIF-1α (−53%), p50 (−74%), and p65 (−60%) expression as compared to the TcdA group. In line with this, pNAPE-LP + palmitate 0.0003 μg/ml also caused a significant recovery of RhoA-GTPase expression (+649%) and a significant inhibition of the systemic release of IL-6 (−86%), NO (−59%), and VEGF (−71%).

### Treatment with pNAPE-LP and Palmitate Improves the Tight Junction Expression of ZO-1 and Occludin in TcdA-Treated WT Mice

As a consequence of TcdA enterotoxicity, immunofluorescence analysis revealed a significant depletion of both ZO-1 and occludin protein expression, key factors regulating colonic mucosa integrity, as demonstrated by a severe loss of relative fluorescent units compared to vehicle.

**FIGURE 5:**
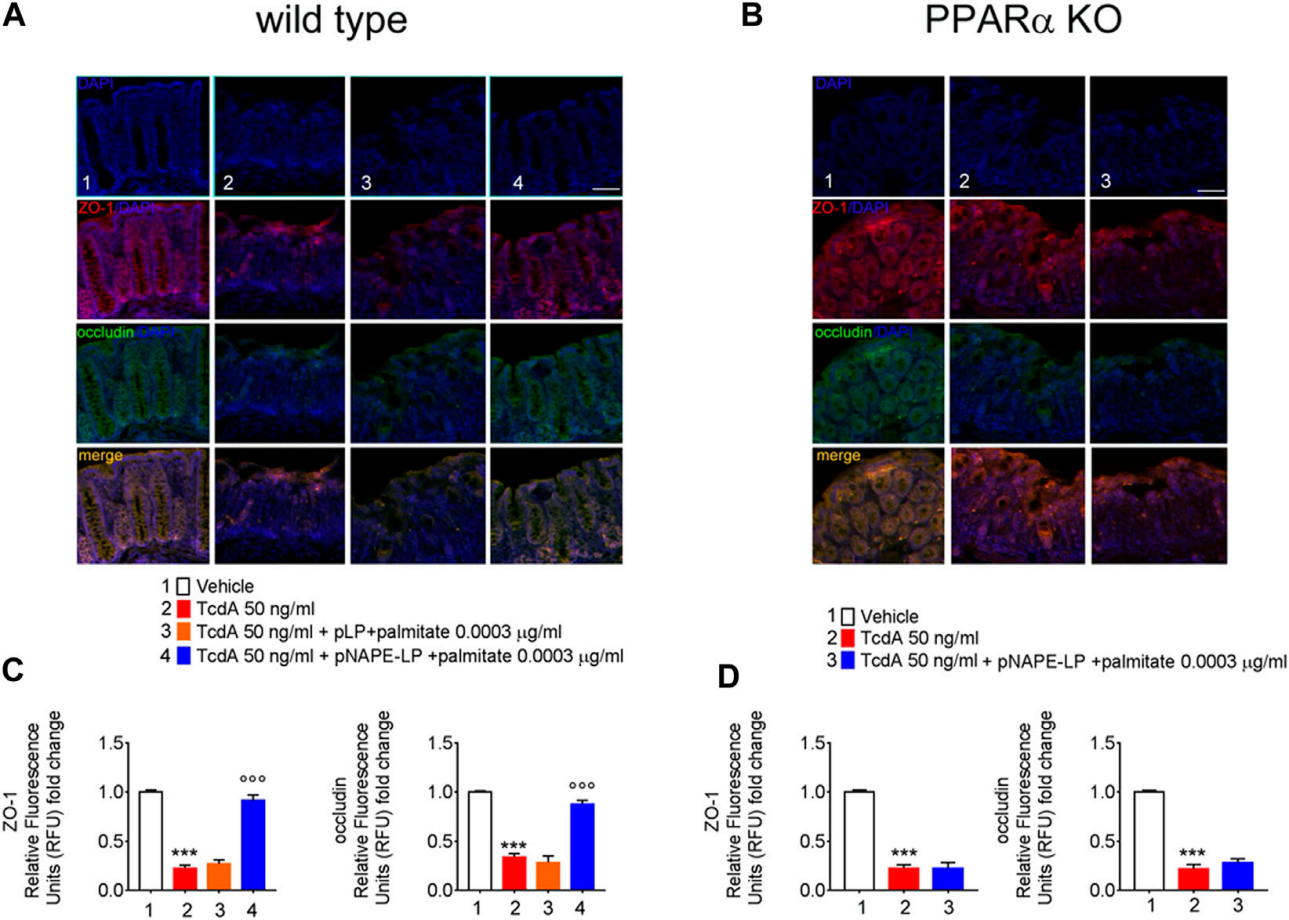
PEA released from pNAPE-LP and ultralow palmitate co-administration restores TcdA-induced colon-barrier disruption, through the upregulation of tight junction proteins ZO-1 and occludin. Representative immunofluorescence images showing the expression of ZO-1 (red), occludin (green), and their merge (yellow) in both wild type **(A)** and PPARα KO **(B)** mice colonic specimen with their respective quantification **(C, D)** showing the PPARα-dependent protective effects of pNAPE-LP combined to palmitate (0.0003 μg/kg) co-administration. Nuclei were also investigated using DAPI staining. Results are expressed as mean ± SEM of *n* = 5 experiments performed in triplicate. ***p < 0.001 vs. vehicle; ^ooo^ *p* < 0.001 vs. DSS-treated mice. Scale bar = 100 μm; magnification 10×. Scale bar: 100 μm.

Specifically, TcdA exposure caused a significant decrease of ZO-1 and occludin in C57BL/6 J mice treated with TcdA (-78% and -77%, respectively vs. vehicle) (Figure 5A). A one-week course of pLP and palmitate did not improve both ZO-1 (+18%) and occludin (-16%) expression vs. TcdA group (Figure 5A), while pNAPE-LP + palmitate 0.0003 μg/ml significantly improved the tight junction protein expression, with a relative increase in fluorescence intensity for both ZO-1 (+304%) and occludin (160%) in WT mice (Figures 5C).

### The Effects of pNAPE-LP and Palmitate are Selectively Mediated by PPARα Receptors

In line with the findings in WT animals, the TcdA challenge caused a significant upregulation of colonic NAPE-PLD expression (+300%) and PEA release (+133%) also in PPARα KO mice. The treatment with pNAPE-LP + palmitate 0.0003 μg/ml caused a significant relative increase in NAPE-PLD tissue expression and PEA release in PPARα KO mice, similarly to what observed in WT animals (+168 and +207%, respectively, compared to TcdA-treated mice (Figures 2B,D,F)).

Nonetheless, despite the increase in PEA tissue levels, the treatment with pNAPE-LP + palmitate 0.0003 μg/ml failed to display all the aforementioned protective effects on the histological damage score and pro-inflammatory markers and tight junction proteins’ expression in this murine model. Indeed, under the same experimental conditions, pNAPE-LP + palmitate 0.0003 μg/ml administration failed to improve the histological damage score (−5%), macrophage density count (+1.5%), and MPO level quantification (+5%) (Figures 3B,F,D,H,J), in spite of the increased tissue production of PEA. Conversely to WT animals, no significant changes were detected neither in the expression of TLR-4 (+13%), RhoAGTPase (+3.7%), phospho-p38 MAPK (-3.7%), HIF-1α (+1.8%), p50 (-6.5%), and p65 (-1.5%) (Figures 4B,D) nor in the plasmatic levels of IL-6 (+3.4%), NO (-7%), and VEGF (+5%) in pNAPE-LP + palmitate 0.0003 μg/ml treated PPARα KO mice vs. the respective TcdA group (Figure 4F), further confirming the role of PPARα receptors in mediating PEA effects. Finally, TcdA exposure caused a significant decrease of ZO-1 and occludin in PPARα KO mice treated with TcdA (−78 and −77%, respectively vs. vehicle), but once again, the rescue of ZO-1 and occludin observed in WT animals treated with pNAPE-LP + palmitate 0.0003 μg/ml appeared to be mediated by PPARα receptors, since both ZO-1 (-0.89%) and occludin (+18%) signal intensity were unmodified by pNAPE-LP + palmitate 0.0003 μg/ml treatment in PPARα KO mice (Figures 5B,D). Taken together, these results suggest the crucial importance of PPARα receptors in mediating the effects of the engineered probiotic pNAPE-LP in our experimental conditions.

## Discussion

With the continuous rise in its incidence and recurrence, there has been an increasing interest toward the development of nonantibiotic-based therapies for *C. difficile* infection. The current treatment guidelines indeed, advise for the use of metronidazole and vancomycin as first-line treatment in CDI (McDonald et al., 2018); however, increasing concerns have been raised regarding the incidence of resistant strains and the rate of recurrence in successfully treated patients (Johnson, 2009). Being broad-spectrum antibiotics themselves, both metronidazole and vancomycin carry the potential to prolong the susceptibility to reinfection, by preventing the replenishment of the resident intestinal microflora and, in so doing, suppressing one of the most important protective colonization resistance factors from the host (Baines and Wilcox, 2015).

In this context, probiotics can, at least on paper, be effective in restoring the intestinal dysbiosis and play a protective role against CDI (Na and Kelly, 2011). Although *Saccharomyces, Bifidobacterium,* and *Lactobacillus* genera all carried a protective effect against *C. difficile* (Mills et al., 2018; Liu et al., 2020), the efficacy of probiotics for CDI prevention and/or treatment is currently limited. In keeping with this, we observed only a modest and not a significant effect in limiting the histopathological damage and regulating the epithelial tight junction protein expression of ZO-1 and occludin, following preventive administration of pLP in our murine model.

Aside from the obvious implication of regulating the host–microbiota imbalance, however, probiotics could serve as delivery systems of anti-inflammatory molecules able to limit CDI severity. Genetically engineered probiotics able to colonize and *in situ* express anti-inflammatory mediators could overcome some of the therapeutic failings in CDI. Feasibility of oral therapy against CDI by means of using engineered *Lactobacillus* able to express toxin-neutralizing antibodies was previously explored in a hamster model and showed therapeutic potential by reducing colonic inflammation and prolonging animals’ survival (Andersen et al., 2016). In the current study, we explored the efficacy of an orally administered pNAPE-LP, in order to achieve an *in situ* delivery and release of PEA in the gastrointestinal tract, under the boost of ultralow doses of exogenous palmitate.

Our results show that pNAPE-LP was an effective strategy to produce PEA, both *in vitro* and *in vivo*. PEA is an endogenous bioactive lipid amide with pleiotropic homeostatic properties, including immune response regulation and inhibition of pain and inflammation, through the activation of PPARα receptors (Sarnelli et al., 2016b). These well-established immunomodulatory properties have been studied in a number of animal and human models featured by hyper-inflammation, such as osteoarthritis (Britti et al., 2017), neurodegenerative, (Beggiato et al., 2019) and, notably, in inflammatory bowel diseases (Pesce et al., 2018).

In our model, the prophylactic oral administration of pNAPE-LP was able to limit the severity of TcdA toxin-induced colitis by improving colonic mucosa histopathological damage and reducing the release of pro-inflammatory mediators in both colonic mucosa and plasma. The increase in PEA tissue levels was followed by a significant downregulation of p50 and p65, markers of NF-κB activation, a key signaling pathway involved in downstream regulating cytokine and intestinal pro-inflammatory mediators’ release (Wullaert, 2010). Besides, neo-angiogenesis induced by the TcdA challenge *in vivo* has been recently reported as a contributing factor in CDI pathogenicity (Huang et al., 2019). PEA release by pNAPE-LP + palmitate 0.0003 μg/ml was followed by a significant reduction of HIF-1α expression, through the inhibition of NF-κB signaling pathway. Since it has been demonstrated that HIF-1α activation prompts a rapid worsening of CDI pathology and mortality (Huang et al., 2019), our results highlight the importance of the control of the neo-angiogenesis as a further protective mechanism of pNAPE-LP administration. This is confirmed by the mitigation of both NO and VEGF levels in our experimental conditions, that is, in line with the previous observations of an antiangiogenic effect of PEA in colon inflammatory conditions (Sarnelli et al., 2016a).

Among the different tight junction proteins, ZO-1 and occludin are key regulators in paracellular permeability in epithelial cells and TcdA is known to mediate its effects on tight junction structure and function via inactivation of Rho proteins (Nusrat et al., 2001). In parallel, PEA release improved, at a protein level, the expression of tight junction proteins ZO-1 and occludin, on one hand and induced a significant rescue of RhoA GTPase, on the other. Although we did not evaluate mucosal permeability, these combined effects likely suggest a marked improvement in epithelial barrier integrity mediated by pNAPE-LP treatment.

In animal models of ulcerative colitis, PEA has previously showed therapeutic potential in improving the histopathological and clinical features of ulcerative colitis. In this models, PEA anti-inflammatory effects were also closely related to the specific reduction of enteric glial cells (EGCs) activation during colitis, mediated by the selective targeting of the S100B/TLR4 axis (Esposito et al., 2014). EGCs mediate a key role in the maintenance of gut homeostasis ensuring the correct trophism of neurons in the enteric nervous system (Fornai et al., 2018), modulating oxidative stress, controlling epithelial barrier functions, and actively participating in intestinal inflammation, by acting as antigen presenting cells (Capoccia et al., 2015). Although exploring the exact pathways involved in TcdA-induced inflammation was beyond the purpose of the present study, one could speculate that pNAPE-LP beneficial effects could also be mediated by PEA ability to counteract EGCs activation, as demonstrated in different models of colitis (Borrelli et al., 2015; Ippolito et al., 2015).

Additionally, PEA is known for its “entourage effect” on the endocannabinoid system (ECS), being able to potentiate the effect of prototypical endocannabinoids, but not carrying their potential side effects (Davis et al., 2019). Interestingly, the non-psychotropic cannabinoid cannabidiol (CBD) was able to prevent the cytotoxic damage caused by TcdA *in vitro* cultured Caco-2 cells (Gigli et al., 2017). The observed increase in mucosal integrity and reduced cellular permeability in this study were mediated by the involvement of the cannabinoid-1 (CB-1) receptor. CBD is a very low-affinity CB1 ligand, that can nonetheless still affect CB1 receptor activity and the ECS *in vivo* in an indirect manner. Although it has been suggested that CBD is well tolerated and safe in humans at high doses and with chronic use, *in vitro* and *in vivo* studies showed potential drug metabolism interactions, cytotoxicity, and decreased CB receptor activity (Bergamaschi et al., 2011; Iffland and Grotenhermen, 2017).

On the contrary, PEA offers the prospect of modulating the ECS without any virtual side effects, owing to its inability of activating the CB receptors (Ambrose and Simmons, 2019). PEA belongs to the Autacoid Local Injury Antagonist (ALIA) amides family, a group of shortly lived lipids that is produced on demand and rapidly metabolized to their inactive metabolites (Okamoto et al., 2004). Since orally administered PEA has an unfavorable pharmacokinetic profile that could prevent an efficient therapeutic response in humans, many murine models use intraperitoneal administration of PEA at high doses in order to achieve its therapeutic effects (Gabrielsson et al., 2016). Hence, one main limiting factor to its clinical transability in humans is PEA often-unpredictable tissue concentrations following oral administration. Here, we demonstrated the feasibility of integrating into the murine microbiota, a genetically engineered probiotic, able to topically biosynthesize PEA, overcoming such limitations.

We used the *L. paracasei subsp. paracasei* F19, a widely used probiotic in clinical settings (Di Cerbo and Palmieri, 2013), that is, featured by its peculiar genetic stability and its ability to colonize and persist in the human intestine (Higl et al., 2007). Analogously to PEA, *L. paracasei subsp. paracasei* F19 is considered safe for human consumption, and during human trials, it showed the absence of adverse effects, even in subjects with underlying disorders, adding to the safety of our system.

One limitation of the current study is that NAPE-PLD gene is a key enzyme responsible for the production of several other bioactive lipids, including oleoyl-ethanolamine (OEA) and anandamide (AEA) and PEA production is often coupled by a relative increase of these bioactive compounds. Although we did not test their levels in our model, the evidence that the protective effects of pNAPE-LP were abolished in PPARα KO mice supports the idea that PEA release is the key factor in mediating such effects. Nonetheless, these so-called entourage effects are not to be excluded a priori when considering the potential therapeutic effects of pNAPE-LP.

Another potential setback is that we explored an acute intestinal disease model by intrarectally injecting mice with TcdA enterotoxin, with a relatively short timeframe between colitis induction and animals’ euthanasia. This model has been chosen because it is highly reproducible and well validated and replicates the main pathological findings of CDI. Nonetheless, being a murine model of acute intestinal injury, the study design prevents us from drawing any definitive conclusions on the effects of pNAPE-LP in terms of microbiota modifications and/or chronic PEA effects following prolonged administrations. This study, indeed, was designed to provide proof of concept data demonstrating the clinical feasibility and effectiveness of this newly designed probiotic in CDI and to evaluate for the first time PEA anti-inflammatory effects in TcdA-induced colitis.

Despite these limitations, the results of the present study highlight the safety and effectiveness of pNAPE-LP that, by counteracting mucosal inflammation and restoring the epithelial barrier function, can improve TcdA-induced colitis in mice. Although further research is needed to evaluate the long-term, ecological, and environmental safety of this genetically modified organism in order to translate this approach in humans, this evidence supports, for the first time, the role of PEA and this genetically engineered probiotic in counteracting CDI in mice.

## Data Availability

The raw data supporting the conclusions of this article will be made available by the authors, without undue reservation, to any qualified researcher
